# Supplementary material for: Modeling Friedreich’s ataxia with Bergmann glia-enriched human cerebellar organoids
Source: bioRxiv. 2025 May 16:2025.05.16.654315. Preprint. [Version 1] doi: 10.1101/2025.05.16.654315 (PMC12132547; doi:10.1101/2025.05.16.654315)

bioRxiv preprint doi: https://doi.org/10.1101/2025.05.16.654315; this version posted May 16, 2025. The copyright holder for this preprint (which was not certified by peer review) is the author/funder. This article is a US Government work. It is not subject to copyright under 17 USC 105 and is also made available for use under a CC0 license.

Figure S1. Immunohistochemical analysis of hCBOs

(A, B) Expression of typical markers of early CPNE development. Note the more widespread expression of R1 region marker EN2 along with the specification of VZ (SOX2) vs SVZ (PAX2, SKOR2). (C) Distribution of CPNE (PAX2) and RL (PAX6, ATOH1) across consecutive sections of the same organoid. All the data in (A)-(C) were obtained from day 35 hCBOs.

Figure S2. Machine learning-based image analysis to track neuronal migration

(A) Image-based analysis to quantify the length of the NeuN+ granule cell layer. The unit is shown in pixels.

(B) Identification of calbindin+ Purkinje cells.

(C) Identification of NeuN+ granule cells.

(D) Identification of DAPI+ cell nuclei.

(E) Box and Whisker plot showing the location of individual calbindin+ Purkinje cells relative to the cortex of the organoid.

(F) Box and whisker plot showing the location of individual NeuN+ granule cells relative to the cortex of the organoid.

The NeuN+ granule cells (masked green in (A) and red in (B)), calbindin+ Purkinje cells (masked green in (C)), and DAPI$^+$ cell nuclei (masked blue in (D)) were identified using an optimized CellPose deep learning method. Upper and lower length regions containing NeuN+ cells were

bioRxiv preprint doi: https://doi.org/10.1101/2025.05.16.654315; this version posted May 16, 2025. The copyright holder for this preprint (which was not certified by peer review) is the author/funder. This article is a US Government work. It is not subject to copyright under 17 USC 105 and is also made available for use under a CC0 license.

defined by the Super Pixel + Graph Cuts machine learning method and highlighted with yellow lines in (A) to quantify the length of the NeuN+ containing cell layer. Using the same method, the upper region of $NeuN^+$ or $calbindin^+$ cells (highlighted as yellow) and cortex of the organoid (highlighted as cyan lines) were defined in (B)-(D). 1 pixel equals 0.4151329 µm in (A), (E), and (F).

Figure S3. Characterization of iPSC lines and hCBOs from health and FRDA iPSC lines

(A) Expression of pluripotency-associated markers OCT4, NANOG, and SOX2 in iPSCs from a healthy individual (GM25256) and two FRDA patients.

(B) Immunohistochemical analysis showing normal hCBO generation and expression of typical cerebellar markers at day 35. No apparent differences were observed between health and FRDA iPSC lines.

(C) Western blot analysis showing primordial cerebellar markers (GBX2 for caudal region of mid-hindbrain boundary, BARHL1 for RL, and PAX2 of CPNE) expressed by corrected, isogenic GM23913 iPSC clones at day 35 compared to day 0.

Figure S4. Ultrastructural analysis of mitochondria in normal and FRDA iPSC lines

(A, B) Higher magnification of electron microscopic images showing normal mitochondria (A) and mitochondria with pathological changes in FRDA (B). See also Figure 6J and 6K.

Figure S5. Characterization of isogenic gene-corrected FRDA iPSC lines

bioRxiv preprint doi: https://doi.org/10.1101/2025.05.16.654315; this version posted May 16, 2025. The copyright holder for this preprint (which was not certified by peer review) is the author/funder. This article is a US Government work. It is not subject to copyright under 17 USC 105 and is also made available for use under a CC0 license.

(A) Expression of pluripotency markers in two established cell clones of gene-corrected FRDA iPSC lines (GM23913).

(B-C) Visualization of FXN intron 1 targeted locus with integrated genome viewer (IGV) in the gene-corrected isogenic iPSCs from a FRDA patient (GM23913). PacBio long-read sequencing of the iPSC clones confirms excision in the FXN intron 1 locus (1244 bp + GAA repeats).

Figure S6. Analysis of the 4 highest ranked off-target genomic sites in the corrected, isogenic GM23913 iPSC clones.

For 5' and 3' gRNA utilized for the genomic deletion, the corrected isogenic GM23913 iPSC clones showed no off-target deletions in any of the predicted off-target sites. The PAM sequences are highlighted in the red box.

Figure S7. Copy number frequency plots of the corrected, isogenic GM23913 iPSC clones identified by optical genome mapping.

Plots showing typical copy number frequency through the entire chromosomes, i.e. 2 copy numbers in chromosomes 1-22 and 1 copy numbers in chromosome x and y, respectively.

was not certified by peer review) is the author/funder. This article is a US Government work. It is not subject to copyright under 17 USC 1
and is also made available for use under a CC0 license.

Figure S1 (Ryu et al.)

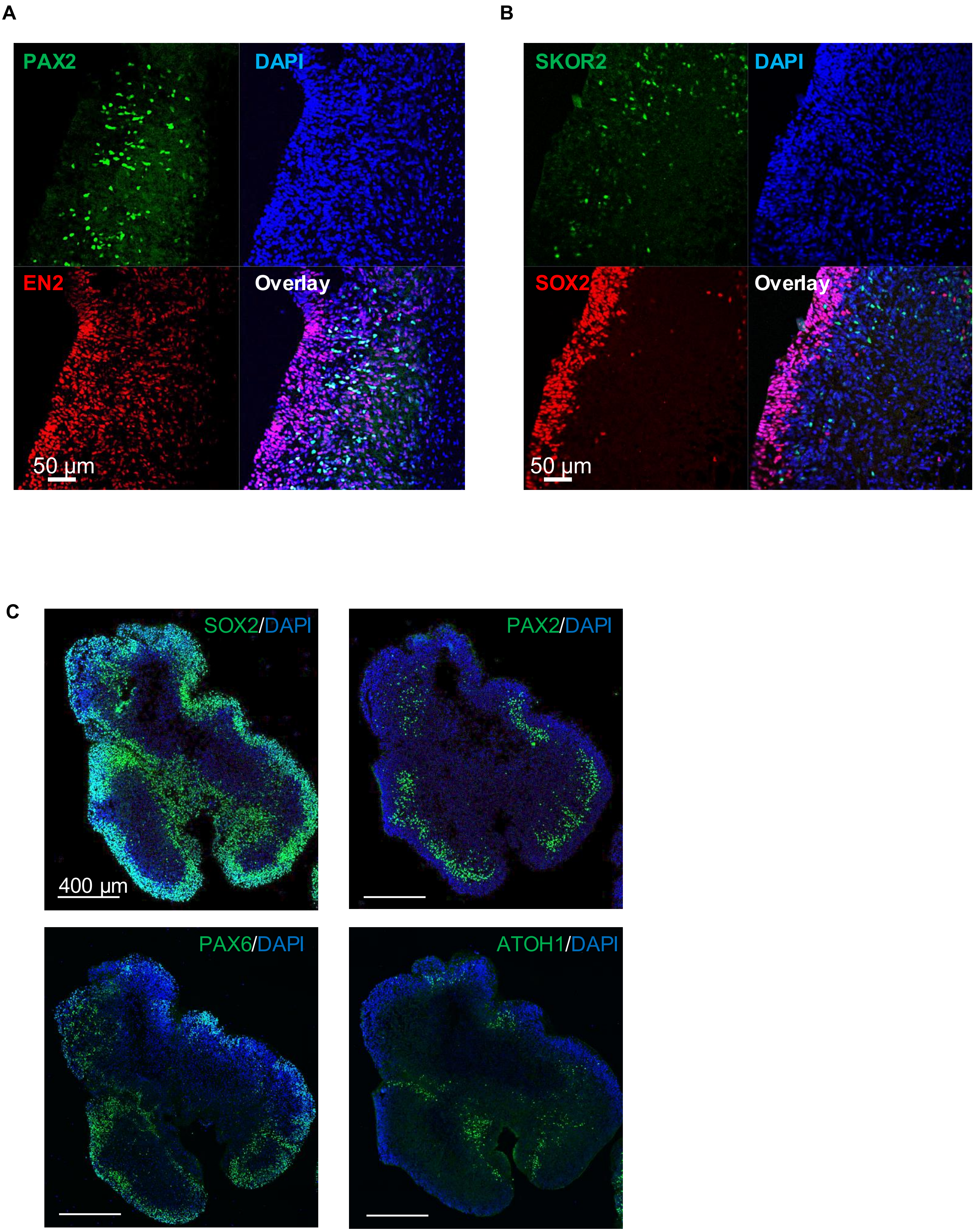

was not certified by peer review) is the author/funder. This article is a US Government work. It is not subject to copyright under 17 USC 1
and is also made available for use under a CC0 license.

Figure S2 (Ryu et al.)

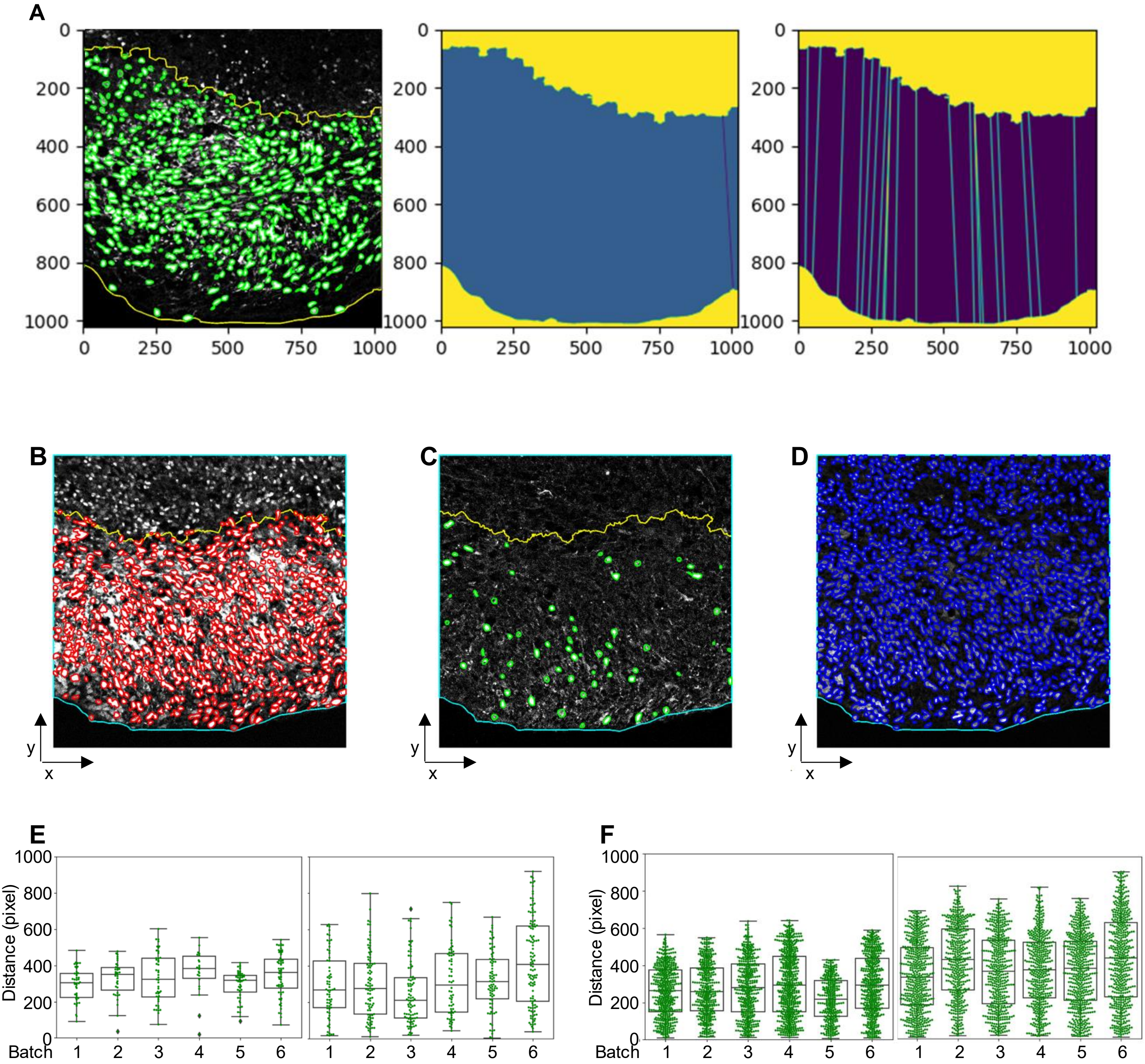

was not certified by peer review) is the author/funder. This article is a US Government work. It is not subject to copyright under 17 USC 1
and is also made available for use under a CC0 license.

Figure S3 (Ryu et al.)

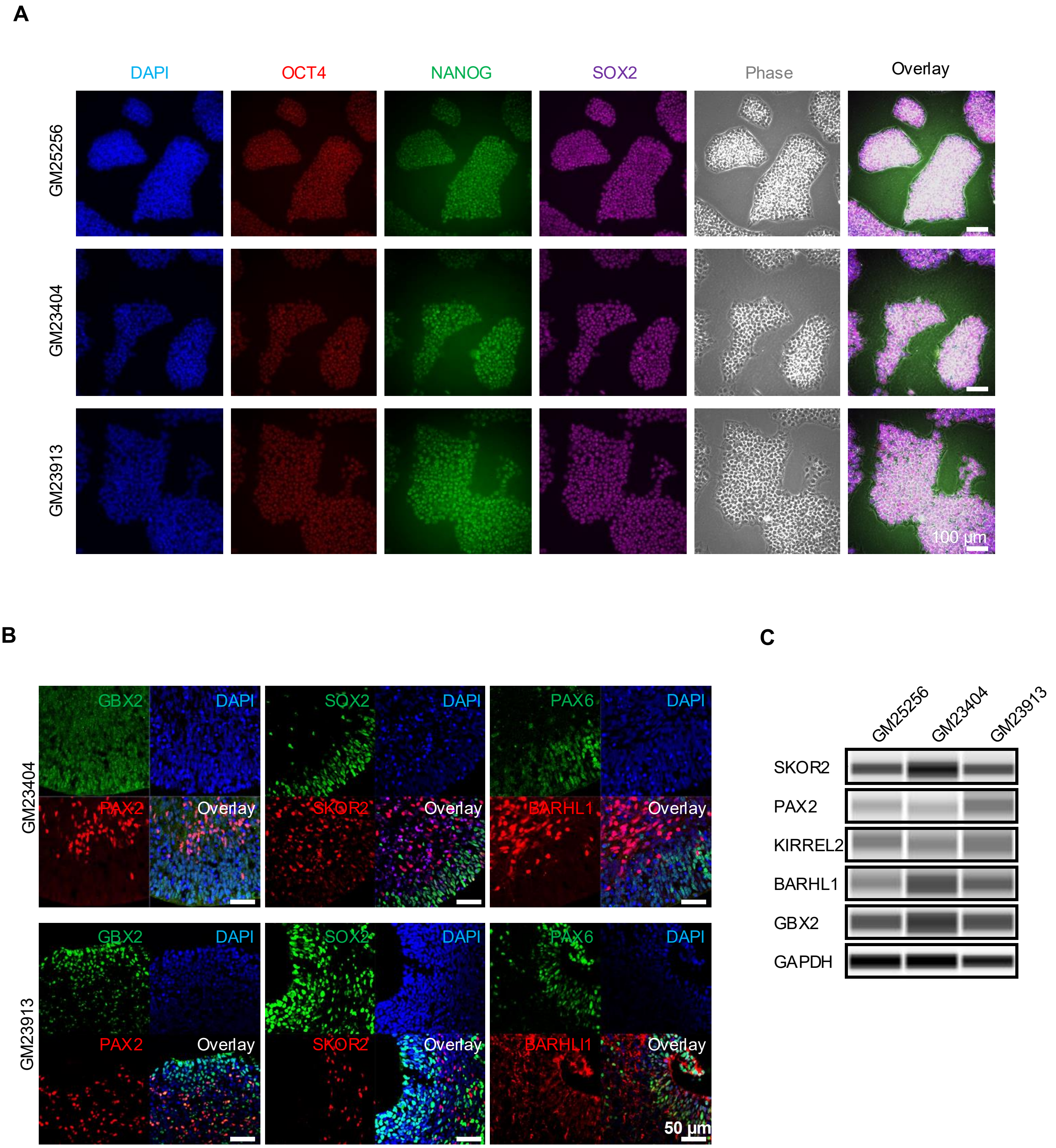

was not certified by peer review) is the author/funder. This article is a US Government work. It is not subject to copyright under 17 USC 1
and is also made available for use under a CC0 license.

Figure S4 (Ryu et al.)

A

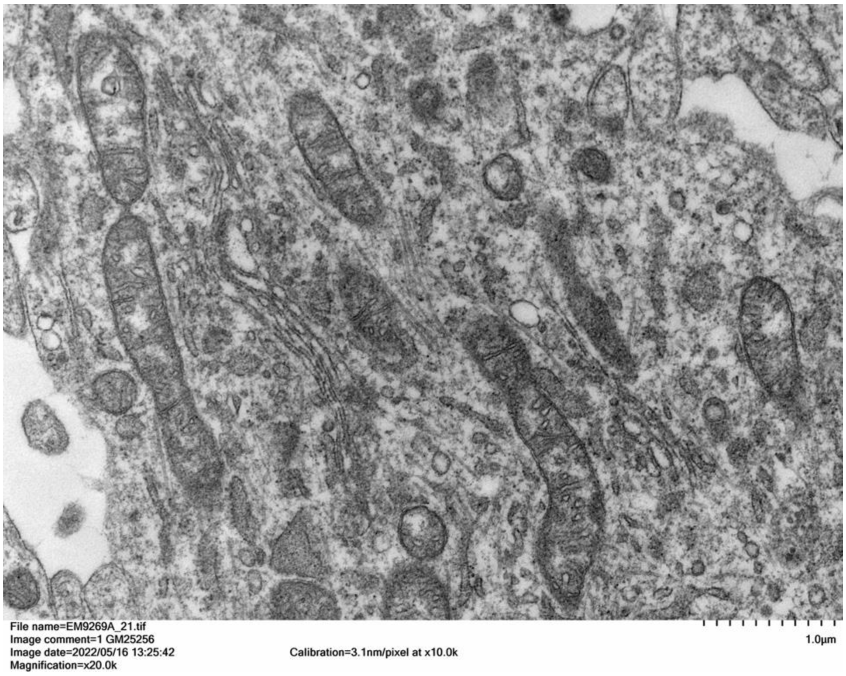

B

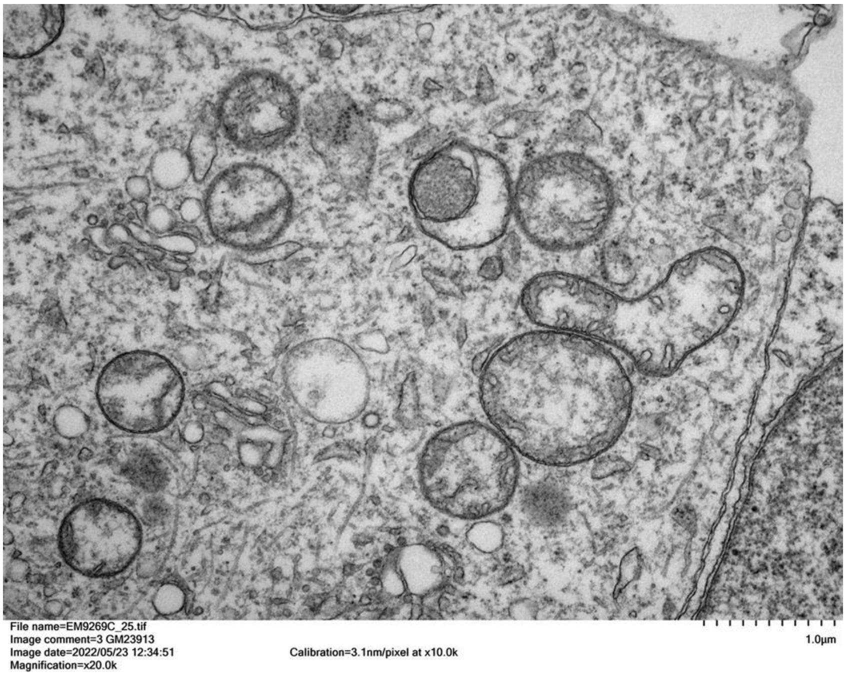

was not certified by peer review) is the author/funder. This article is a US Government work. It is not subject to copyright under 17 USC 1
and is also made available for use under a CC0 license.

Figure S5 (Ryu et al.)

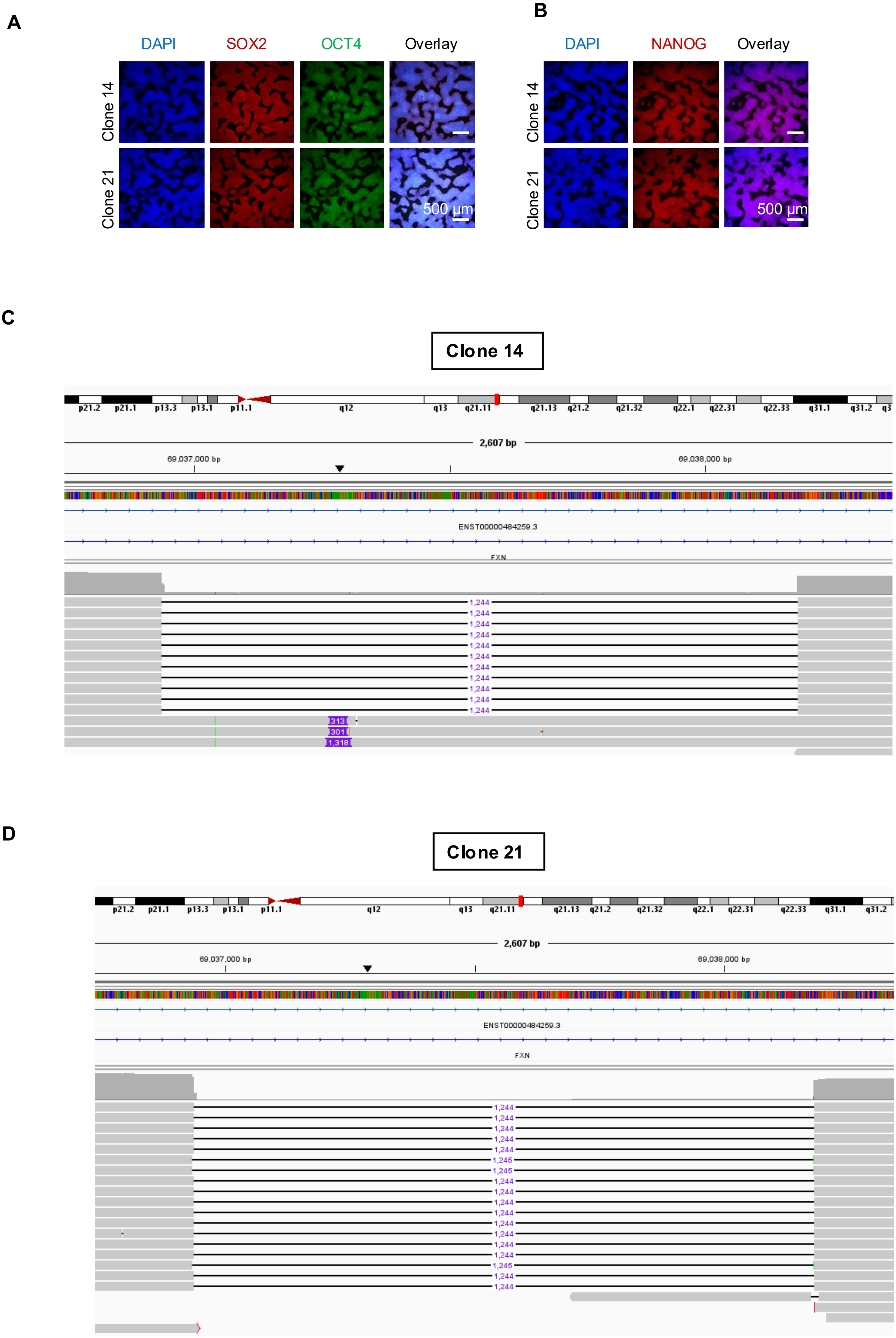

was not certified by peer review) is the author/funder. This article is a US Government work. It is not subject to copyright under 17 USC 1
and is also made available for use under a CC0 license.

Figure S6 (Ryu et al.)

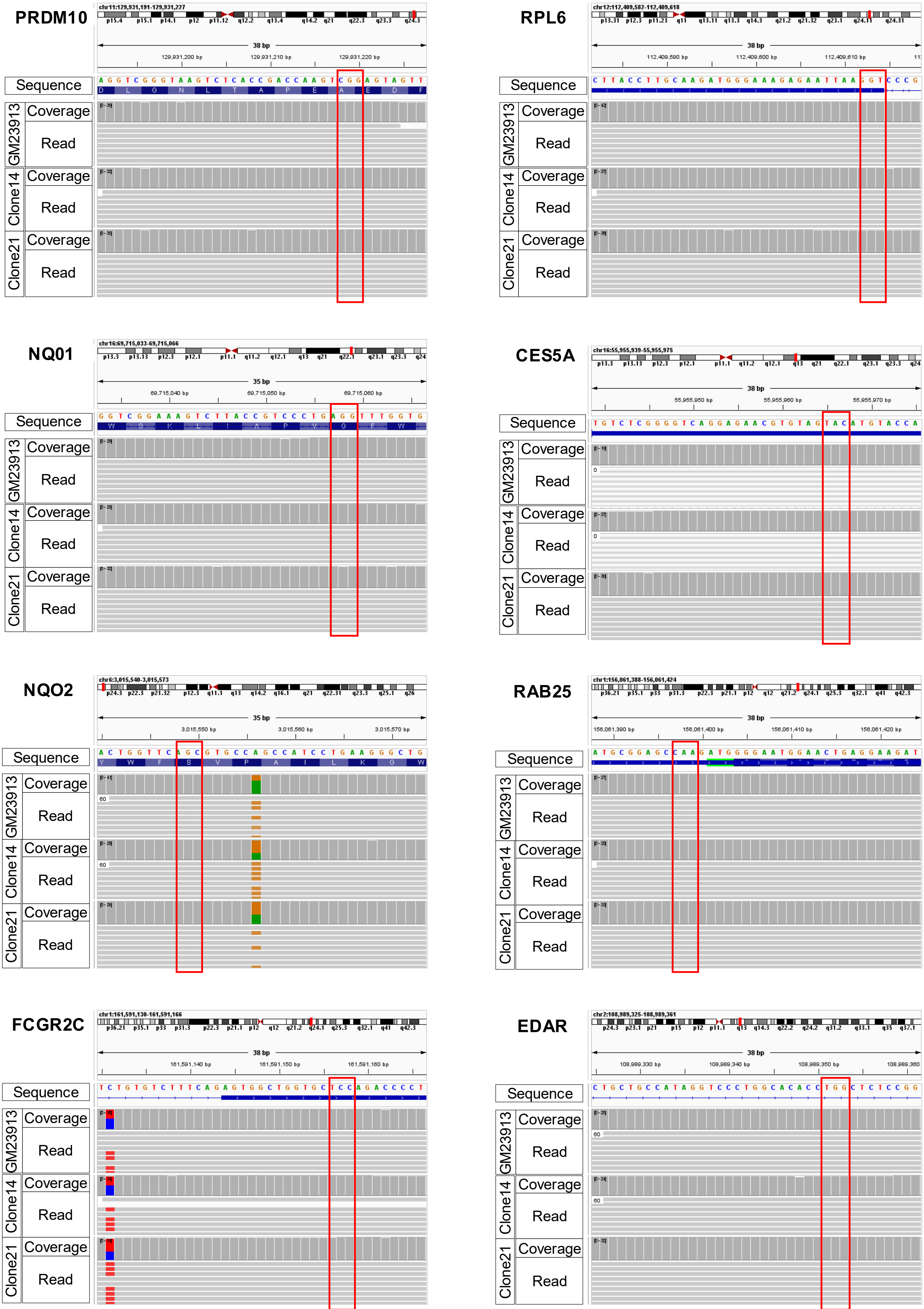

was not certified by peer review) is the author/funder. This article is a US Government work. It is not subject to copyright under 17 USC 1
and is also made available for use under a CC0 license.

Figure S7 (Ryu et al.)

Clone 14

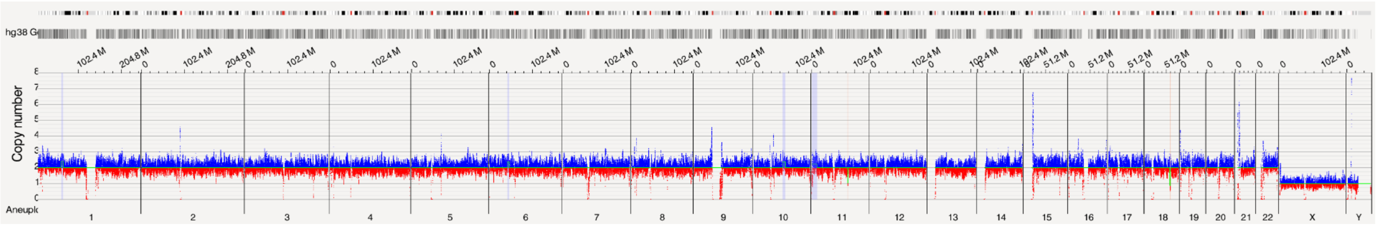

Clone 21

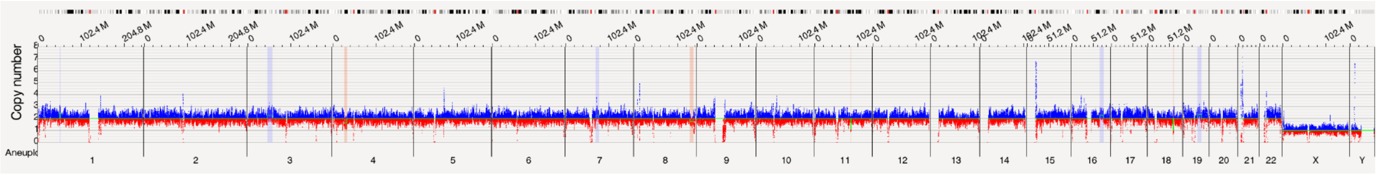

Supplement: 1 [file NIHPP2025.05.16.654315V1-supplement-1.pdf]
